# Supplementary material for: Differential binding cell-SELEX method to identify cell-specific aptamers using high-throughput sequencing
Source: Sci Rep. 2019 May 31;9:8142. doi: 10.1038/s41598-019-44654-w (PMC6544647; doi:10.1038/s41598-019-44654-w)
Supplement: Supplementary file 1 — Supplementary data [file 41598_2019_44654_MOESM1_ESM.pdf]

Differential binding cell-SELEX method to identify cell-specific aptamers using high-throughput sequencing

Karlis Pleiko[1]*, Liga Saulite[1], Vadims Parfejevs[1], Karlis Miculis[2], Egils Vjaters[2] and Una Riekstina[1]

[1] Faculty of Medicine, University of Latvia, Riga, LV-1004, Latvia

[2] Pauls Stradins Clinical University Hospital, Riga, LV-1002, Latvia

* To whom correspondence should be addressed. Tel: +371 28680049; Email: karlis.pleiko@lu.lv

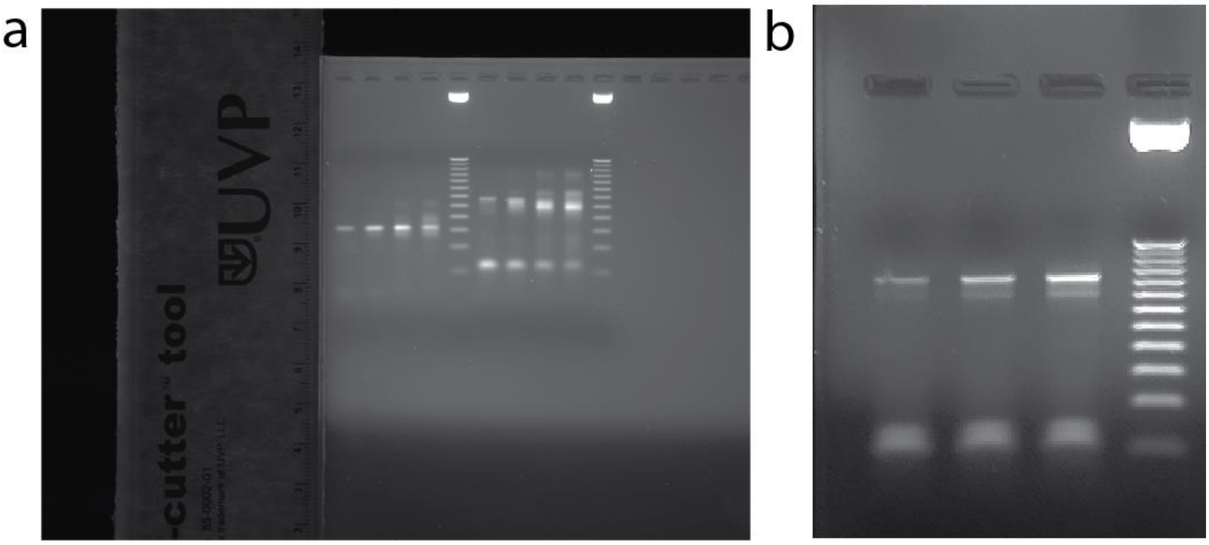

Figure S1. Gel pictures for 1st overhang and 2nd overhang products. a – first four wells from left side contain PCR product amplified using standard primers without overhang, fifth well contains 25 bp size marker, sixth to ninth well contain 1st overhang products amplified using different number of PCR cycles that are comparable to first four wells, 10th well contains 25 bp size marker. Cycle number was chosen based on less byproducts formed (sixth well). b – first three wells contain 2nd overhang products using different concentrations, fourth well contains 25 bp size marker. Cycle number was chosen based on most product observed (third well). Images were acquired using built in UVP software and combined using Adobe Illustrator.

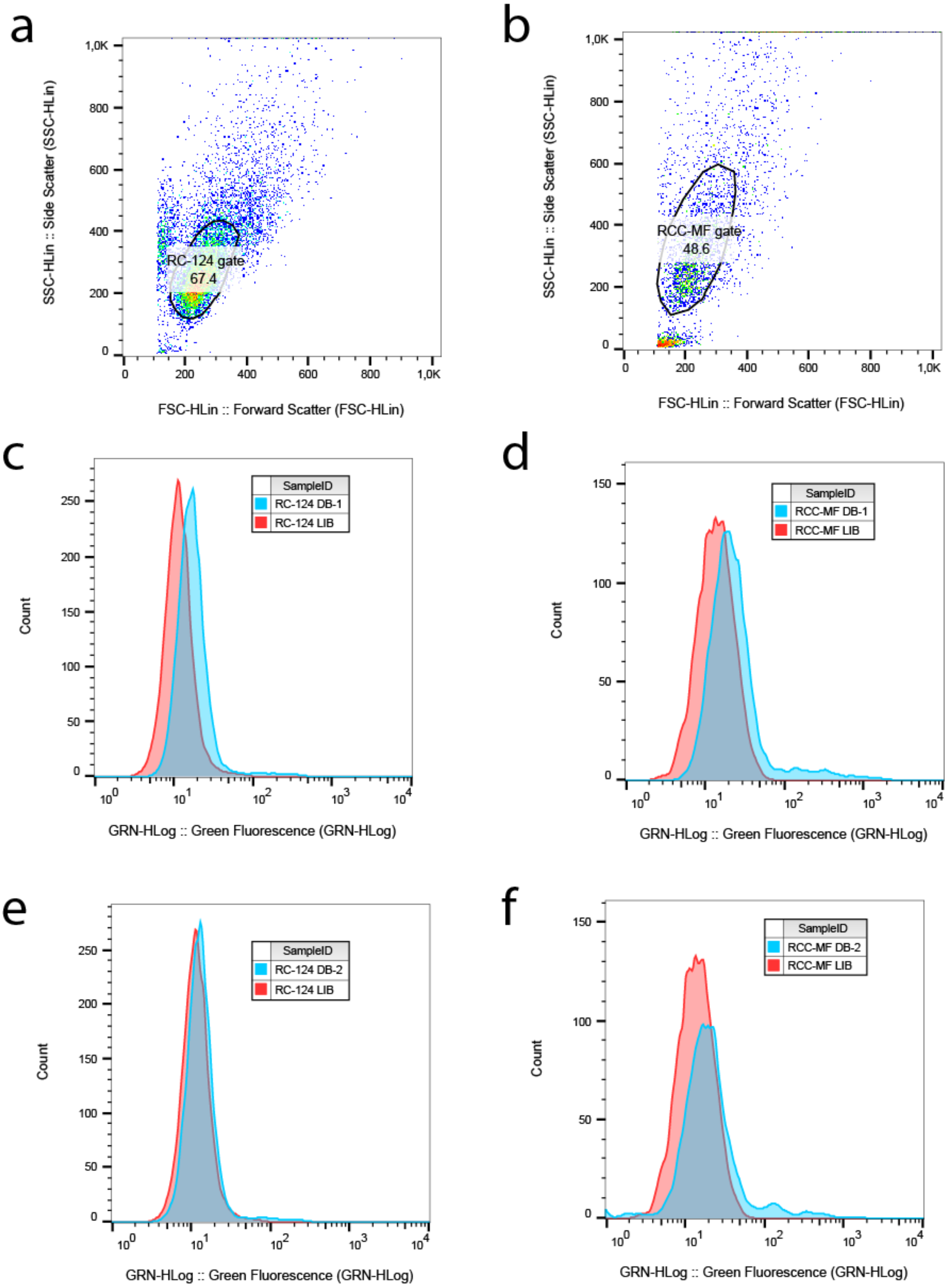
a
RC-124 gate
67.4
SSC-HLin :: Side Scatter (SSC-HLin)
FSC-HLin :: Forward Scatter (FSC-HLin)
1,0K
800
600
400
200
0
b
RCC-MF gate
48.6
SSC-HLin :: Side Scatter (SSC-HLin)
FSC-HLin :: Forward Scatter (FSC-HLin)
c
SampleID
RC-124 DB-1
RC-124 LIB
Count
GRN-HLog :: Green Fluorescence (GRN-HLog)
d
SampleID
RCC-MF DB-1
RCC-MF LIB
Count
GRN-HLog :: Green Fluorescence (GRN-HLog)
e
SampleID
RC-124 DB-2
RC-124 LIB
Count
GRN-HLog :: Green Fluorescence (GRN-HLog)
f
SampleID
RCC-MF DB-2
RCC-MF LIB
Count
GRN-HLog :: Green Fluorescence (GRN-HLog)

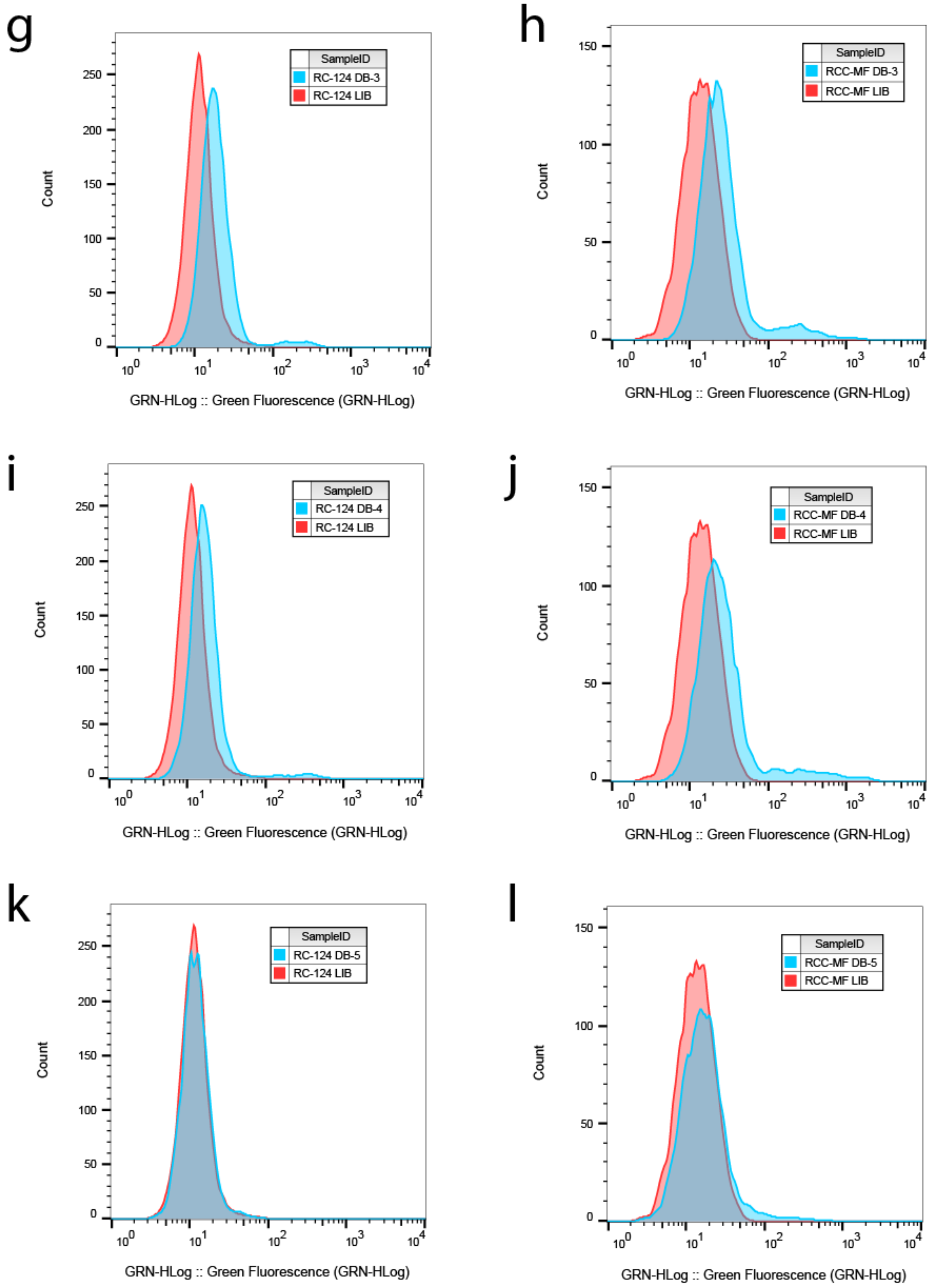
g
h
i
j
k
l
Count
GRN-HLog :: Green Fluorescence (GRN-HLog)
SampleID
RC-124 DB-3
RC-124 LIB
RCC-MF DB-3
RCC-MF LIB
RC-124 DB-4
RCC-MF DB-4
RC-124 DB-5
RCC-MF DB-5

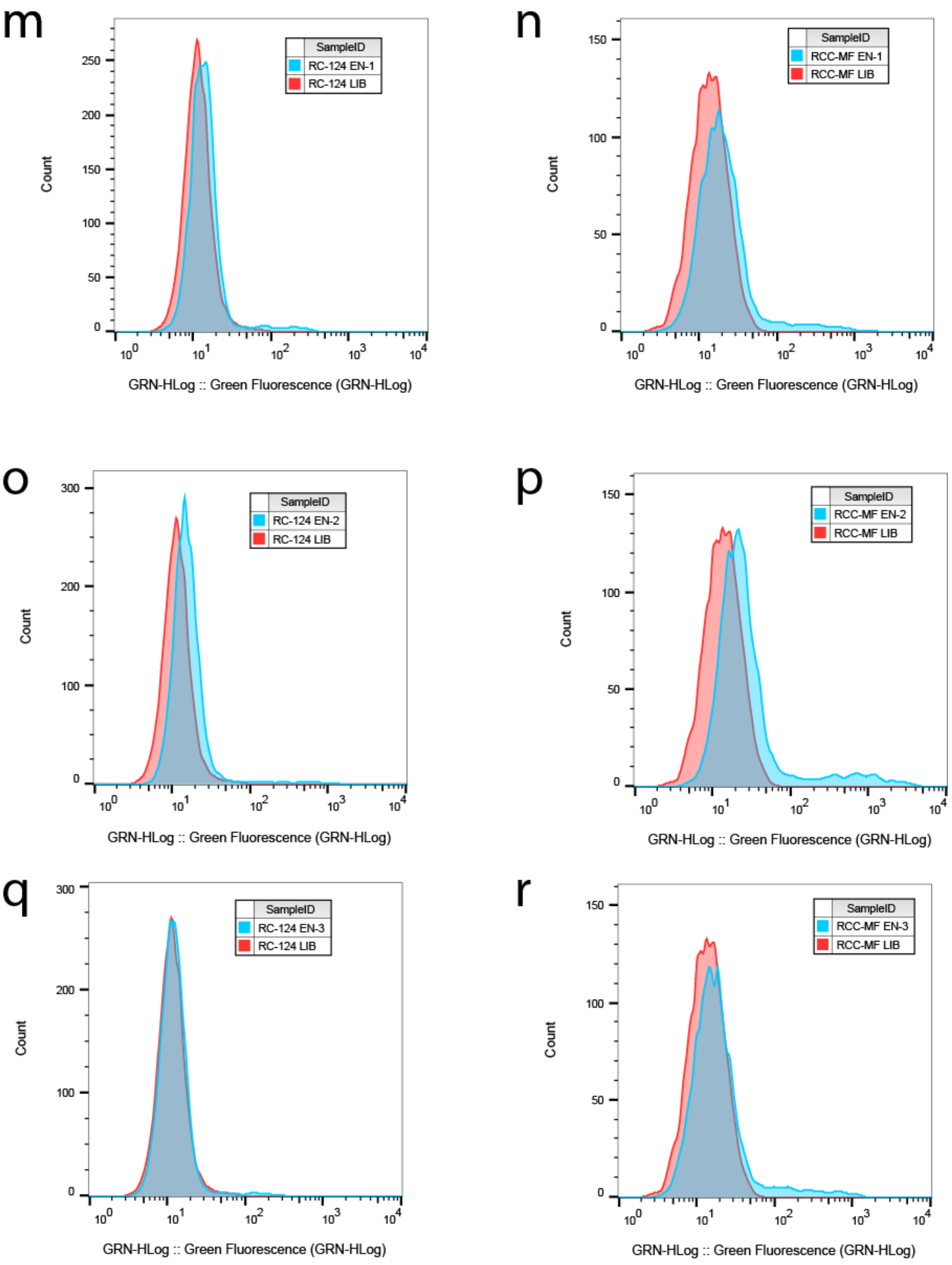
m
Count
GRN-HLog :: Green Fluorescence (GRN-HLog)
SampleID
RC-124 EN-1
RC-124 LIB
n
Count
GRN-HLog :: Green Fluorescence (GRN-HLog)
SampleID
RCC-MF EN-1
RCC-MF LIB
o
Count
GRN-HLog :: Green Fluorescence (GRN-HLog)
SampleID
RC-124 EN-2
RC-124 LIB
p
Count
GRN-HLog :: Green Fluorescence (GRN-HLog)
SampleID
RCC-MF EN-2
RCC-MF LIB
q
Count
GRN-HLog :: Green Fluorescence (GRN-HLog)
SampleID
RC-124 EN-3
RC-124 LIB
r
Count
GRN-HLog :: Green Fluorescence (GRN-HLog)
SampleID
RCC-MF EN-3
RCC-MF LIB

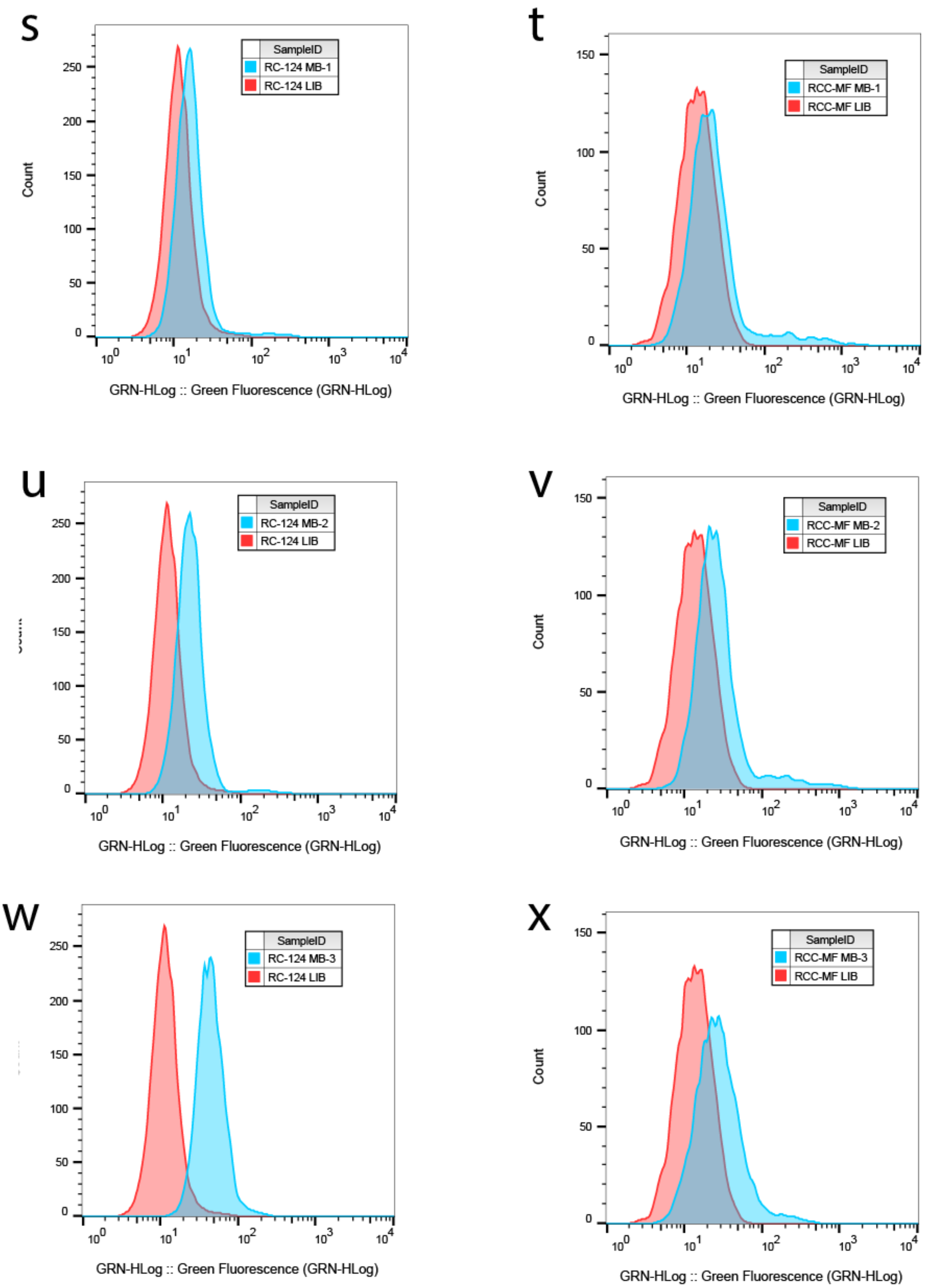

Figure S2. Representative flow cytometry histograms for Figure 7. Gating of RC-124 negative control (a) and RCC-MF target (b) cells. DB-1 sequence binding to RC-124 (c) and RCC-MF (d). DB-2 sequence binding to RC-124 (e) and RCC-MF (f). DB-3 sequence binding to RC-124 (g) and RCC-MF (h). DB-4 sequence binding to RC-124 (i) and RCC-MF

(j). DB-5 sequence binding to RC-124 (k) and RCC-MF (l). EN-1 sequence binding to RC-124 (m) and RCC-MF (n). EN-2 sequence binding to RC-124 (o) and RCC-MF (p). EN-3 sequence binding to RC-124 (q) and RCC-MF (r). MB-1 sequence binding to RC-124 (s) and RCC-MF (t). MB-2 sequence binding to RC-124 (u) and RCC-MF (v). MB-3 sequence binding to RC-124 (w) and RCC-MF (x).

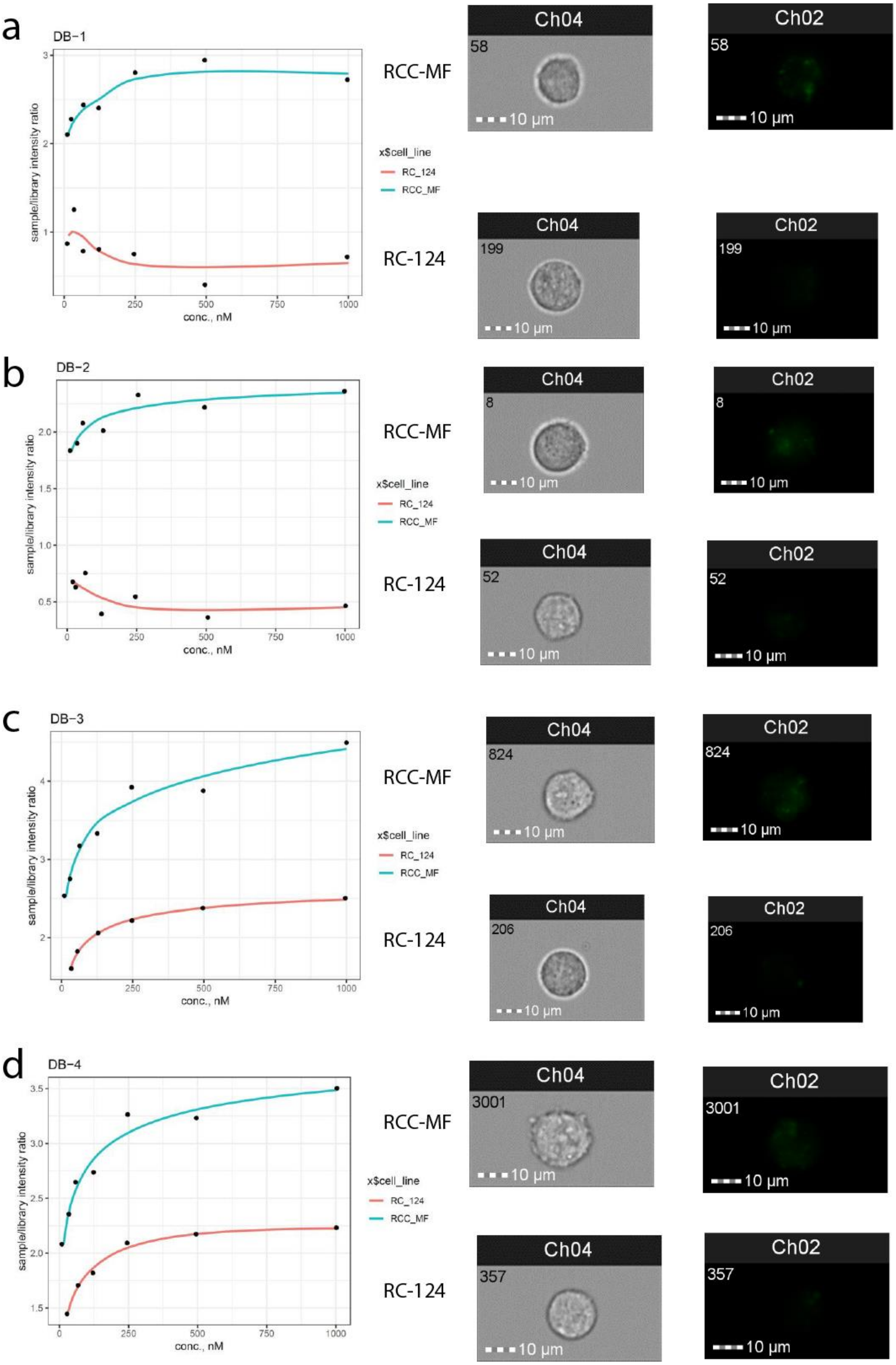
a
DB−1
sample/library intensity ratio
conc., nM
x$cell_line
RC_124
RCC_MF
RCC-MF
RC-124
Ch04
Ch02
58
199
10 µm
b
DB−2
8
52
c
DB−3
824
206
d
DB−4
3001
357

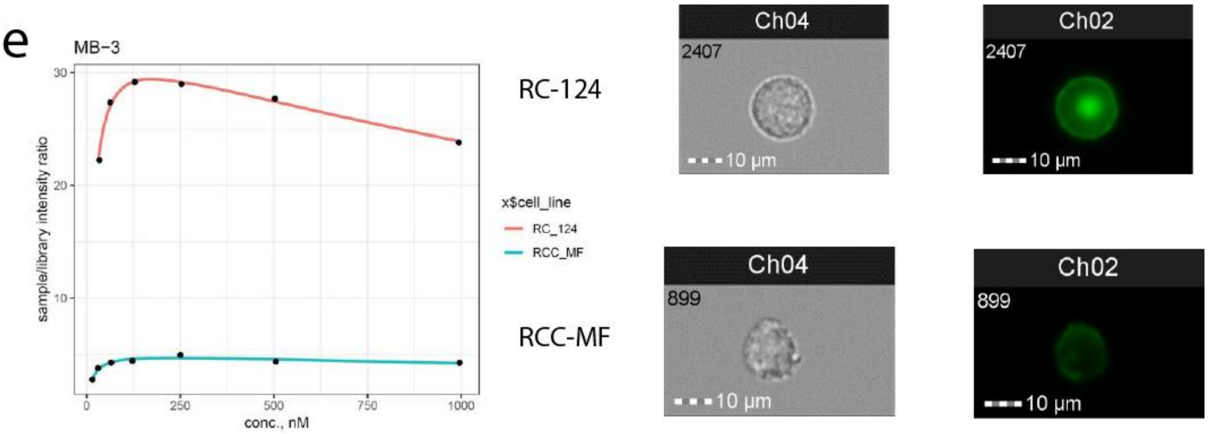

Figure S3. Detailed comparison for data in Figure 8. Concentration dependent binding of sequences DB-1 (a), DB-2 (b), DB-3 (c), DB-4 (d), MB-3 (e) to RCC-MF target and RC-124 negative control cells.
